# Detection of genetic variation and activity analysis of the promoter region of the cattle tRNA-modified gene *TRDMT1*

**DOI:** 10.5194/aab-64-147-2021

**Published:** 2021-04-30

**Authors:** Xiaohua Yi, Shuai He, Shuhui Wang, Haidong Zhao, Mingli Wu, Shirong Liu, Xiuzhu Sun

**Affiliations:** 1 College of Animal Science and Technology, Northwest A&F University, Yangling, Shaanxi, 712100, PR China; 2 College of Grassland Agriculture, Northwest A&F University, Yangling, Shaanxi 712100, PR China

## Abstract

The tRNA modification gene in eukaryotes is relatively conservative. As an important modification gene, the *TRDMT1* gene plays an important role in maintaining tRNA structural maintenance and reducing mistranslation of protein translation by methylation of specific tRNA subpopulations. Mouse and zebrafish *TRDMT1* knockout experiments indicate that it may mediate growth and development through tRNA modification. However, there are no systematic reports on the function of tRNA-modified genes in livestock. In this study, Qinchuan cattle DNA pool sequencing technology was used. A G>C mutation in the -1223 bp position upstream of the *TRDMT1* translation initiator codon was found. At this locus, the dual-luciferase assay indicated that different genotypes cause differences in transcriptional activity (P<0.05). Our experiment detected a natural genetic variation of a tRNA modification gene *TRDMT1*, which may provide potential natural molecular materials for the study of tRNA modification.

## 1 Introduction

China has a wealth of local cattle breeds, of which the top five cattle are representatives of high-quality cattle breeds, namely Qinchuan cattle, Jinnan cattle, Nanyang cattle, Luxi cattle and Yanbian cattle. Beef cattle mainly provide humans with high-protein beef products. With the improvement of people's living standards, the demand for beef and other products is also increasing. Hence, the improvement of beef cattle breeds is urgent. At present, gene-editing methods can be modified at the level of nucleobase modification, including DNA, histones, RNA, etc. which may all be useful in beef cattle breeding (Vojta et al., 2016).

RNA modification is a common phenomenon in molecular biology. Various types of RNA in cells can be modified after transcription. Many chemical modifications are conservative, suggesting that RNA modification is related to protein translation (Grosjean et al., 2014). In biology, tRNA is a key factor in the transition between mRNA and protein. The maturation of tRNA requires the splicing of introns and chemical modification of specific loci to mature (Hopper and Phizicky, 2003). Some tRNA-modified genes are associated with metabolic defects, including CDK5-like regulatory subunit-related protein 1 (CDKAL1), tRNA aspartate methyltransferase 1 (*TRDMT1*) and tRNA methyltransferase 10 homolog A (TRMT10A) (Sarin and Leidel, 2014). *TRDMT1*, as an RNA methyltransferase known to methylate tRNA, is recruited to DNA damage sites and required for the induction of RNA m5C (Kunert et al., 2003; Jurkowski et al., 2008; Rai et al., 2007; Chen et al., 2020). Knockout experiments confirmed that it weakly modified animal and plant DNA, but there is no significant difference between the knockout type and wild type in *Drosophila*, *Arabidopsis* and mice (Goll et al., 2006). Subsequently, it was found that *TRDMT1* protein can form 5-methylcytidine (m5C) on tRNA and mRNA. Tuorto et al. (2012) found that the double-knock-type mouse embryonic fibroblasts have reduced proliferative capacity, and at the same time, protein synthesis is restricted. Xue et al. (2019) found that knockdown of *TRDMT1* significantly inhibited HEK293 cell proliferation and migration but had no effect on clonogenic potential. The inhibitory effects could be attenuated by re-expression of *TRDMT1* in HEK293 cells.

*TRDMT1* is also known as Dnmt2, the most conserved member of the DNA methyltransferase family, which has been shown to methylate tRNAs (Goll et al., 2006). As a member of the epigenetic modification factor, *TRDMT1* can both methylate genomic DNA and modify RNA. It has mostly been characterized as either targeting tRNA or rRNA and can be chemically modified for specific tRNA subpopulations in different eukaryotes (Schumann et al., 2020; Sibbritt et al., 2013; Bohnsack et al., 2019). Epigenetic modification is an important reason for the spatiotemporal expression of genes and also plays a key role in the growth and development of animals. The knockdown of *TRDMT1* expression caused a decrease in the level of tRNA modification, and the development of animal bones, muscles and other tissues was limited, suggesting that its expression may affect the relevant life processes.

The specific modification of tRNA ensures the correct assembly of tRNA during protein translation (Pütz et al., 1994), avoiding erroneous loading. tRNA-specific modifications can maintain the stability of tRNA, and the lack of necessary modifications may lead to premature decay of tRNA, shortening its half-life period (Alexandrov et al., 2006). tRNA modification can also stabilize its structure and enhance nuclease tolerance (Schaefer et al., 2010). In addition, tRNA modification can maintain its structural and functional stability.

The occurrence of tRNA modification affects life activities by affecting the synthesis of proteins. Liu et al. (2015) reported that the DNA chemical modification factor DNMT family gene SNP locus was associated with corpus callosum mass, lean meat color and flank thickness. The tRNA modification gene is evolutionarily conserved, but most of the research exists only in the model organism such as yeast and mouse. The state of tRNA modification is associated with disease, growth and metabolism (Sarin et al., 2014; Rai et al., 2007; Barrett er al., 2008; Cătoi et al., 2015). Vitamin B12 and folic acid levels in pregnant women with *TRDMT1* mutations are significantly different from those in the wild-type population, and this gene polymorphism is associated with the occurrence of congenital spina bifida in the fetus (Franke et al., 2009).

In this experiment, by detecting the mutation in the promoter region of the bovine *TRDMT1* gene, fluorescent recombinant plasmids of different genotypes were constructed at this site, and the relative fluorescence intensity was analyzed by detecting the transfected 293T cells. The binding and influence of the mutation site and the transcription factor were analyzed by software. Meanwhile, we detected the relative expression of *TRDMT1* gene in each tissue. This study provides a certain theoretical basis for the study of livestock *TRDMT1* gene expression on its life activities and the study of tRNA modification in animal life processes.

## 2 Materials and methods

### 2.1 DNA extraction

A total of 224 Qinchuan cattle (2–6 years old) were collected in this research. All selected individuals were healthy and unrelated. All DNA was obtained from the blood samples by phenol chloroform (Pang et al., 2011).

### 2.2 DNA pool construction

All DNA samples were diluted to working concentration (50 ng/µL) according to previous report by Li et al. (2013). Three groups of 30 individuals per group were randomly composed, and each group of samples was uniformly mixed into one tube. After shaking, the mixture was centrifuged to form a DNA pool, which was used as templates for polymerase chain reaction (PCR) amplification.

### 2.3 Primer design, PCR protocol and DNA sequencing

The 5${}^{\prime }$ flanking region sequences of *TRDMT1* gene were downloaded from Ensembl (http://asia.ensembl.org/index.html, last access: 20 August 2019). As shown in Table 1, we designed a total of five pairs of primers to scan the *TRDMT1* gene including the first exon and the 5${}^{\prime }$ flanking region totaling 1468 bp. The PCR program was set to ensure that a sufficient number of the target fragments were amplified: predegeneration at 95 ${}^{\circ }$C for 5 min, followed by 35 cycles of denatured at 95 ${}^{\circ }$C for 30 s, annealed at 55/57 ${}^{\circ }$C for 30 s, and extended at 72 ${}^{\circ }$C for 30 s, finally extended at 72 ${}^{\circ }$C for 10 min. PCR amplification was performed using bovine mixed-pool DNA as a template, and specific identification was performed by 2.5 % agarose gel electrophoresis. Then, the products were sequenced only when each pair of primers showed a single objective band.

**Table 1:** TRDMT1 promoter genetic variation detection primers.

Fragment	Sequences (5${}^{\prime }$–3${}^{\prime }$)	$T_{m}$	Product
		(${}^{\circ }$C)	size (bp)
1TRDMT1QD	F: ACTGTGCATCAGGCATGTGA	57	393
	R: TCCTGGGTACACTAGAGGGC		
2TRDMT1QD	F: CTGCCCTGTGAAGACCTGAG	57	280
	R: TAGTTCCGCGGCTTTTCAGT		
3TRDMT1QD	F: ACTCAAGCTAAGGCCCAACC	57	339
	R: CTCAGGTCTTCACAGGGCAG		
4TRDMT1QD	F: TTGGAGAAGGAAGGCCACAG	55	422
	R: GACACTGTGCATCAGGCATG		
5TRDMT1QD	F: GACCATTTCTGCTCCTCCC	–	1468
	R: GCCCTGTACCGTCTCACCT		

### 2.4 Product purification and vector transform

We used the fifth pair of primers to amplify the *TRDMT1 *promoter region using the Qinchuan cattle mixed pool as a template (Table 1). The complexity of the promoter base motif leads us to only obtain non-specific products. SanPrep Column DNA Gel Extraction Kit was used to purify the target sequence. PMD-19T vector was used to link purification and enrichment products. Mutations were introduced using the sixth and seventh pairs of primers (Table 2). Similarly, both wild-type and mutant sequences were ligated to the T vector and transformed using the DH5α competent state. The plasmids were extracted using the omega kit. All methods were performed according to the protocol.

**Table 2:** TRDMT1 promoter introduced mutation primer.

Primer	Sequences (5${}^{\prime }$–3${}^{\prime }$)	$T_{m}$ (${}^{\circ }$C)
Ftrdmt1gC	F1: cta*GCTAGC*GACCATTTCTGCTCCTCCC	64
	R1: GAGGCTATGGGGGAAGAGGTC	
Ltrdmt1gC	F2: GACCTCTTCCCCCATAGCCTC	64
	R2: ccc*AAGCTT*GCCCTGTACCGTCTCACCT	

### 2.5 Digestion reaction and plasmid construction

NheI and HindIII restriction endonucleases were used to digest the pGL3-Basic plasmid; we get the wild type product plasmid and the mutant product plasmid after digestion. The digestion products were purified and ligated into pGL3-Basic plasmid with Solution I.

### 2.6 Cell transfection and transfection

Human embryonic kidney (HEK) 293T cell was cultured in Dulbecco's modified Eagle's medium (DMEM) high-glucose medium containing 10 % fetal bovine serum (FBS) (Gibco, USA), supplemented with 100 units/mL penicillin, 0.1 mg/mL streptomycin, and incubated at 37 ${}^{\circ }$C in 5 % CO${}_{2}$. The cells were passaged every 1 to 2 d. Before transfections, cells were seeded into 24 well plates at a density of $1\times 10^{4}$ cells per well and incubated 1 to 2 d. When the cell density was 80 %, it was replaced with antibiotic-free and serum-free DMEM/F12 medium (Gibco, USA) and incubated for 6 h. The experiment was divided into four groups: A, B, C and D, which were transfected with pGL3-Control, pGL3-Basic, WT and Mut plasmids, respectively, with three replicates in each group. Transfection medium was prepared by mixing 4 µL Lipofectamine 2000 with 0.8 µg transfected plasmid and 10 ng pRL-TK plasmid in 0.5 mL OPTI-MEM medium (Gibco, USA) and incubating the mixture for 20 min at room temperature. Transfection was carried out by substituting 0.5 mL from the DMEM/F12 medium covering the cells with the transfection mix. After 12 h, the transfection medium was removed, covered with 1 mL OPTI-MEM medium.

### 2.7 Dual-luciferase reporter gene assay

According to a report by Derikx et al. (2015), 48 h after transfection, cells were rinsed with PBS. Relative luciferase activity was measured using Synergy H1 (BioTek, USA).

### 2.8 Tissue expression profiling test

RNA was extracted from different tissues of Qinchuan cattle by the TRIzol method. The cDNA was obtained by reverse transcription using the PrimeScript RT kit (TaKaRa, Kusatsu, Shiga Prefecture, Japan), and the concentration was controlled to a uniform 50 ng/µL.

Primers of the *TRDMT1* mRNA expression test were designed using Beacon Designer 8.14 software (Premier Biosoft International, Palo Alto, CA, USA), and glyceraldehyde-3-phosphate dehydrogenase (GAPDH) was used as an internal reference gene (Table 3). The reaction contained 100 ng of cDNA, 10 µL SYBR^®^ Premix Ex Taq TM II (TaKaRa, Japan) and 10 pmol of primers in a volume totaling 20 µL. The mixture was denatured for 30 s at 95 ${}^{\circ }$C and was followed by 40 cycles of 5 s at 95 ${}^{\circ }$C and 30 s at 60 ${}^{\circ }$C.

**Table 3:** The primers used for qPCR analysis.

Primer	Sequences (5${}^{\prime }$–3${}^{\prime }$)	$T_{m}$
		(${}^{\circ }$C)
GAPDH	F1: CACCCTCAAGATTGTCAGCA	56
	R1: GGTCATAAGTCCCTCCACGA	
TRDMT1	F2: TTTAATGAGCCCACCCTGTCA	56
	R2: TGTCCTTGGATCAGTCACATCA	

### 2.9 Statistical analysis

Independent sample t test was used to evaluate the relative the statistical significance of the differences in wild type and promoter variant of *TRDMT1* gene.

P<0.05 was considered significant. Based on the amplification efficiency of the target gene and the reference gene, according to the CT value obtained by qRT-PCR, a group close to the average value was selected as the control group. Then, the relative expression level was calculated using 2${}^{-\Delta \Delta \mathrm{Ct}}$. The GraphPad Prism 8.0 software (GraphPad Software Inc., San Diego, CA, USA) was used for the analysis.

## 3 Results

### 3.1 Genetic variation detection and introduction of promoter mutations of *TRDMT1* promoter

**Figure 1:**
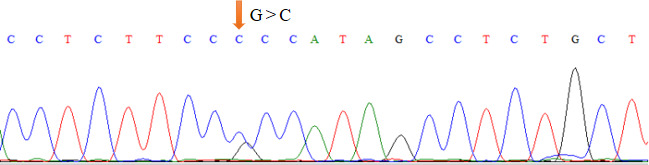
*TRDMT1* promoter genetic variation site.

**Figure 2:**
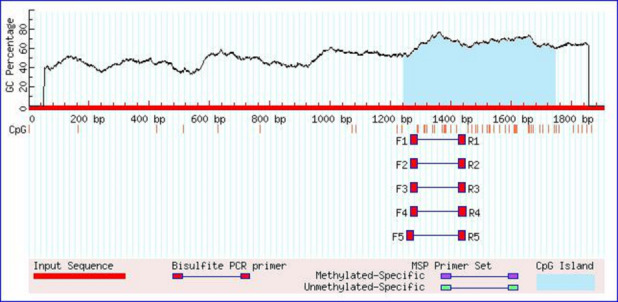
Prediction result of the CpG island in the *TRDMT1* promoter region.

As shown in Table 1, the union set of the sequencing regions of the five pairs of primer amplification products was detected. We first discovered the G>C mutation located upstream of the cattle *TRDMT1* translation initiator codon (Fig. 1). The transcription initiation site was predicted using the Promoter 2.0 Prediction Server software (http://www.cbs.dtu.dk/services/Promoter/, last access: 10 May 2019). The results showed that the -1216 was the transcription initiation site. Methylation island prediction (Li and Dahiya, 2002) showed that there was a methylation island between the first exon and -725 (Fig. 2), suggesting that this position may be involved in the regulation of gene expression. We have set up gradient PCR, but unfortunately the fifth pair of primers in Table 1 can only produce non-specific amplification products.

### 3.2 Predicting promoter variation and transcription factor binding difference

**Figure 3:**
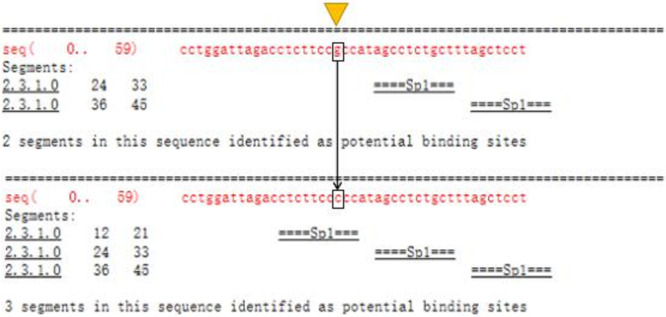
TRANSFAC predicts differences in promoter variation and transcription factor binding.

**Figure 4:**
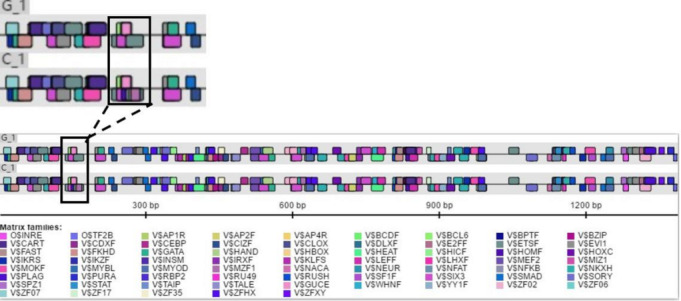
Genomatix predicts differences in promoter variation and transcription factor binding.

The Ftrdmt1gC and Ltrdmt1gC amplification products were diluted 50-fold respectively and used as a template. Ftrdmt1gC F was used as the upstream primer, and Ltrdmt1gC R was used as the downstream primer. After PCR amplification, we obtained the mutant promoter sequence. After vector sequencing, the accuracy of all fragments ligated into the T vector in the experiment was confirmed. The combination of TRANSFAC and Genomatix found that the G>C mutation may cause a difference in binding between the transcription factor Sp1, the pleomorphic adenoma gene (PLAG1), the zinc finger protein (ZNF35) and the bone marrow zinc finger 1 factor (MZF1) transcription factor (Figs. 3 and 4).

### 3.3 Double luciferase activity analysis of *TRDMT1* promoter genetic variation

**Figure 5:**
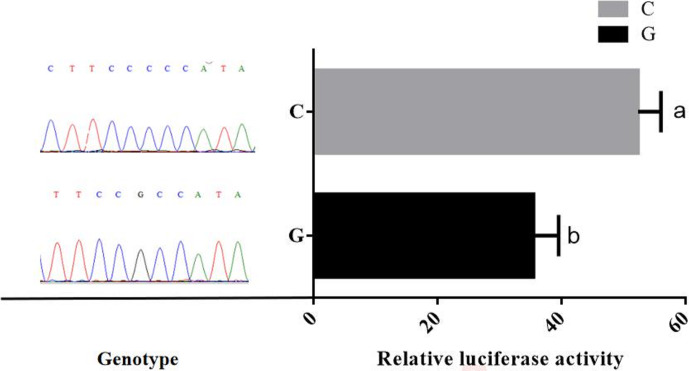
Comparison of relative luciferase activity of *TRDMT1* promoter variant.

Promoter activity of different genotypes of *TRDMT1* was assessed using luciferase reporter gene expression. After calibration by the control group and the basic group, the results showed that the relative activity of the mutant promoter was 1.4 times that of the wild type (P<0.05) (Fig. 5).

### 3.4 The tissue expression profile of *TRDMT1* in Qinchuan cattle

**Figure 6:**
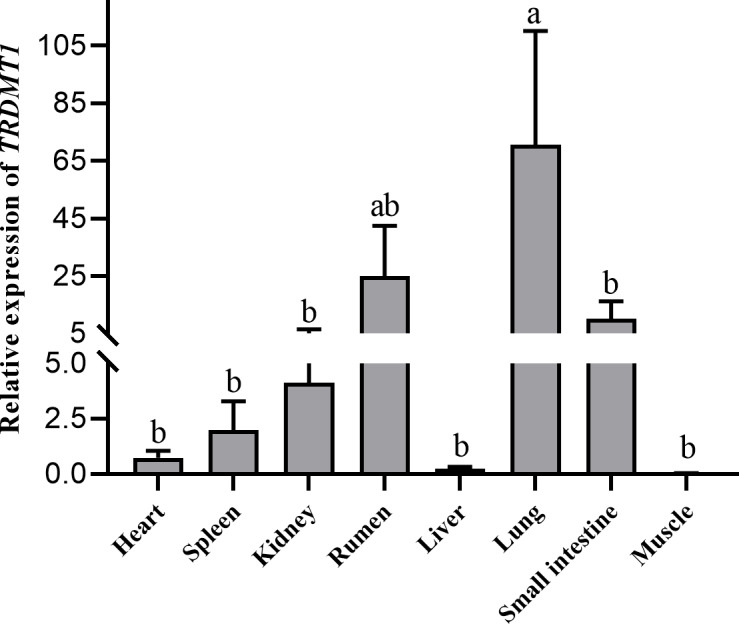
Expression profiling of the *TRDMT1* gene in Qinchuan cattle.

Heart, spleen, kidney, rumen, liver, lung, small intestine and muscle tissues were utilized to detect the expression of the *TRDMT1* gene. The result showed the different expression levels in each tissue. The result revealed that *TRDMT1* was differentially expressed in the different tissues. Its expression is significantly higher in lung than in other tissues (p<0.01), followed by the highest expression in rumen (p<0.05), and the lowest expression in muscle and liver. There was no significant difference in the expression level among other tissues. the lowest in the fetal cattle, but the difference in calves and adult cattle stage was not statistically significant (p>0.05) (Fig. 6).

## 4 Discussion

As an apparent modifier, *TRDMT1* can chemically modify not only DNA but also a specific tRNA subgroup modifier. The occurrence of modification affects life activities by affecting protein synthesis. Liu et al. (2015) reported that the SNP locus of DNA chemical modifier *DNMT* family gene is related to carcass quality, lean color, flanks thickness and other traits. The *TRDMT1* gene is relatively conserved in the biological evolution process. Although it is named tRNA aspartate methyltransferase 1, Tuorto et al. (2012) constructed an RNA bisulfite sequencing map and found that the mouse *TRDMT1* gene has an effect on tRNA-AspGTC and tRNA. tRNA-ValAAC, tRNA-GlyGCC and tRNA-LeuCAA all have a methyl modification at position C38, and the modified tRNA subgroups may be tRNAs that connect mRNA and protein during the translation of most proteins in life activities. *TRDMT1* gene mediates tRNA through modification self-stability and reduces misreading during protein translation. Gene function verification in mice showed that *TRDMT1* expression is related to embryonic bone development and brain development (Tuorto et al., 2012). In zebrafish gene function verification, *TRDMT1* is related to the development of tissues such as the retina and brain (Rai et al., 2007). The tRNA modification genes are evolutionarily conserved, but most studies only exist at the level of model animals such as yeast and mice, and there is a lack of functional studies on the tRNA modification genes of large animals, such as cattle and sheep.

In this study, initially we used Promoter 2.0 Prediction Server software to predict that the transcription start site of the bovine *TRDMT1* gene is the promoter region -1216. Then we used mixed-pool sequencing to scan the G>C mutation at the promoter region -1223 polymorphic loci. For different genotype sequence models, we used TRANSFAC to predict the binding sites of transcription factors and found that G/C mutation may cause a difference in the binding of basic transcription factor sp1 (Fig. 3). The sp1 transcription factor belongs to the sp protein family and is the most abundant type of transcription factor in cells. Sp1 has a certain preference for binding to GC-rich promoters (Kadonaga et al., 1986). As a nucleoprotein, sp1 expression changes during development, and sp1 knockout mice exhibit embryonic lethality (Letovsky and Dynan, 1989). Sp1 is involved in the regulation of the cell cycle, and its protein level is reduced in senescent cells, and its expression level is also related to some cancers (Oh et al., 2007; Takami et al., 2007; Safe and Abdelrahim, 2005). We speculate that the expression level of sp1 protein in each tissue cell is the same among cattle individuals during the same period. The change of gene motif leads to the deletion of sp1 binding site and may downregulate the gene expression level. Then we used Genomatix to predict transcription factor binding and found the following: G>C mutation may increase the binding of the pleomorphic adenoma gene *PLAG1*, zinc finger protein ZNF35 and the bone marrow zinc finger 1 factor MZF1 transcription factor (Fig. 4), suggesting that the *TRDMT1* gene may also be the target gene regulated by *PLAG1*. Tang et al. (2013) reported that *PLAG1* regulates the expression of IGF2 and affects human embryonic development. The *PLAG1* gene has a 96.4 % homology to humans, suggesting that the *PLAG1* gene structure is similar to humans and may participate in its expression regulation as a potential transcription factor for the *TRDMT1* gene.

The conservation of tRNA-modified gene structures in eukaryotes suggests similar functions and regulatory roles (Hopper and Phizicky, 2003). The effect of the *TRDMT1* gene on zebrafish and mouse development leads us to care about its effects on livestock development (Rai et al., 2007). In yeast experiments, the modified genes produced few phenotypes except for the tRNA anticodon loop region. But the *TRDMT1* gene modifies a specific tRNA subpopulation in the anticodon loop. In addition to mutant construction, the phenotypic effects produced by differences in gene expression levels are also a method of reflecting gene function. Differences in the transcriptional activity of genetic promoter genetic variants can be indirectly identified by dual-luciferase assays. Establishing the relationship between genetic variation and expression was also important for the protection of germplasm resources and the development of genetic resources. Hence, we performed a dual-luciferase assay. We used 293T cells for verification, which has the characteristics of high transfection efficiency and easy culture. However, 293T cells are derived from humans. As an experiment material, it was able to analyze the transcription factors shared by eukaryotic cells, but they did not reflect the endogenous characteristics unique to the cattle. The differential binding of transcription factors showed that the genetic variation of this site caused a difference in the activity of bovine *TRDMT1* promoter, which may cause a difference in the expression level of *TRDMT1*. In addition, we found a correlation between the strong linkage structure of bovine *TRDMT1* exon genetic variation and the growth traits of cattle (data not published). It was suggested that the expression level and structural variation of bovine *TRDMT1* gene may have a potential impact on its growth and development. Although we predicted that the transcriptional factors of the *TRDMT1* promoter may cause differential binding, transfection of different genotypes into 293T cells showed that the variant affected its transcriptional activity at the eukaryotic level, but we were not sure which transcription factors are differentially combined, suggesting that the use of different tissues of the cattle to study the promoter variant structure is important for regulating the expression of the *TRDMT1* gene.

Due to the complexity of the sequence structure of the bovine *TRDMT1* promoter region, the test predicts the promoter region by transcription start site prediction and CpG island position prediction. The promoter truncation vectors were not constructed, and the specific position of the *TRDMT1* gene core promoter was not explored. The dual-luciferase vectors pGL3-Basic (G) and pGL3-Basic (C) were constructed, both of which represent the region of the *TRDMT1* promoter -1387/+81 fragment. Excessive fragments may result in decreased transcriptional activity and regulation of transcriptional repressors. However, the results of the experiment indicated that the G>C mutation at the -1223 bp position upstream of the *TRDMT1* translation initiator codon caused a difference in the transcriptional activity (Fig. 5). When the promoter region of bovine *TRDMT1* gene was in the C genotype, the transcriptional activity of the gene was significantly higher than that of the G genotype. It was suggested that the G>C mutation may affect the binding activity of key transcription factors in the promoter region of bovine *TRDMT1* gene and affect the transcription level of the gene.

In the past, *TRDMT1* gene function studies were restricted to model animals such as mice and zebrafish. The research of *TRDMT1* gene function mainly involved interference, inhibition, knockdown and knockout experiments and did not overexpress the expression of *TRDMT1*. The study of the charge of tRNA modification was lacking. The modification of tRNA and the translation of protein did not establish a comprehensive and accurate correspondence. The related studies did not respond to cell fate led by sufficient or excess tRNA modification. In this experiment, mixed-pool sequencing technology was used to find that there was a G>C mutation in the promoter region -1223 of the yellow cattle *TRDMT1* gene. Fluorescent recombinant plasmids of different genotypes were constructed at this site. The transfected 293T cells were analyzed, and the relative fluorescence intensity was analyzed. The fluorescence intensity is significantly higher than that of wild-type G. Through software analysis, the mutation is located near the transcription start site, and the C allele increases the possibility of the binding of transcription factors such as sp1, PLAG1, ZNF35 and MZF1 and affects the transcriptional regulation of bovine *TRDMT1* and detected the relative expression of *TRDMT1* gene in each tissue. This study provides a theoretical basis for the study of livestock *TRDMT1* gene expression on its life activities and the study of tRNA modification in animal life processes.

## Data Availability

No data sets were used in this article.
